# 2-Bromo-4′-methoxychalcone and 2-Iodo-4′-methoxychalcone Prevent Progression of Hyperglycemia and Obesity via 5′-Adenosine-Monophosphate-Activated Protein Kinase in Diet-Induced Obese Mice

**DOI:** 10.3390/ijms19092763

**Published:** 2018-09-14

**Authors:** Chi-Ting Hsieh, Fang-Rong Chang, Yi-Hong Tsai, Yang-Chang Wu, Tusty-Jiuan Hsieh

**Affiliations:** 1 Graduate Institute of Natural Products, College of Pharmacy, Kaohsiung Medical University, Kaohsiung 807, Taiwan; u98831001@gmail.com (C.-T.H.); aaronfrc@kmu.edu.tw (F.-R.C.); lyph0719@hotmail.com (Y.-H.T.); yachwu@kmu.edu.tw (Y.-C.W.); 2 Department of Marine Biotechnology and Resources, College of Marine Sciences, National Sun Yat-sen University, Kaohsiung 804, Taiwan; 3 Graduate Institute of Medicine, College of Medicine, Kaohsiung Medical University, Kaohsiung 807, Taiwan; 4 Research Center for Environmental Medicine, Kaohsiung Medical University, Kaohsiung 807, Taiwan; 5 Lipid Science and Aging Research Center, Kaohsiung Medical University, Kaohsiung 807, Taiwan

**Keywords:** chalcone, halogen, flavonoid, adipocyte, skeletal muscle

## Abstract

Obesity and diabetes are global health-threatening issues. Interestingly, the mechanism of these pathologies is quite different among individuals. The discovery and development of new categories of medicines from diverse sources are urgently needed for preventing and treating diabetes and other metabolic disorders. Previously, we reported that chalcones are important for preventing biological disorders, such as diabetes. In this study, we demonstrate that the synthetic halogen-containing chalcone derivatives 2-bromo-4′-methoxychalcone (compound **5**) and 2-iodo-4′-methoxychalcone (compound **6**) can promote glucose consumption and inhibit cellular lipid accumulation via 5′-adenosine-monophosphate-activated protein kinase (AMPK) activation and acetyl-CoA carboxylase 1 (ACC) phosphorylation in 3T3-L1 adipocytes and C2C12 skeletal myotubes. In addition, the two compounds significantly prevented body weight gain and impaired glucose tolerance, hyperinsulinemia, and insulin resistance, which collectively help to delay the progression of hyperglycemia in high-fat-diet-induced obese C57BL/6 mice. These findings indicate that 2-bromo-4′-methoxychalcone and 2-iodo-4′-methoxychalcone could act as AMPK activators, and may serve as lead compounds for a new class of medicines that target obesity and diabetes.

## 1. Introduction

Obesity and metabolic syndrome are growing health issues and life-threatening situations worldwide [1,2]. The consequence of obesity can result in many diseases, including impaired glucose tolerance and type 2 diabetes mellitus (T2DM) [1]. The associated complications of diabetes, such as cardiovascular diseases, peripheral vascular diseases, stroke, diabetic neuropathy, amputations, renal failure, and blindness, result in increased disability, reduced life expectancy, and enormous healthcare costs [2]. To date, the major categories of medicines used to treat T2DM generally have adverse effects or problems with tolerability, especially for patients with liver and renal function disorders [3]. Therefore, the discovery and development of new categories of medicines for treating diabetes and metabolic disorders are urgently needed.

Chalcones, a class of open-chain flavonoids, can not only be biosynthesized by plants, but can also be synthetically manufactured [4,5]. Different plant-derived forms of chalcone have been isolated that contain several substitutive patterns, including hydroxy, methyl, and methoxy substituents on both of the aromatic rings [6,7,8,9,10,11]. However, halogen-containing chalcone derivatives are rare in the plant kingdom [5]. Halogen-containing secondary metabolites have attracted recent attention due to their potent biological activities, which are attributed to the unique electronic and steric environment created by the presence of halogen atoms [4]. Using a simple synthetic method, we have been able to produce different halogen-containing chalcone derivatives [6,12].

In our previous study, we synthesized 60 chalcone derivatives and evaluated their antidiabetic effects by measuring their activity in promoting glucose consumption in 3T3-L1 adipocytes. We found that chalcones with chloro, bromo, and iodo substitutions at position 2 on the A-ring exhibited excellent activities in promoting glucose consumption in 3T3-L1 adipocytes [6]. The efficacy of these derivatives was even more potent than that of the antidiabetic drugs pioglitazone and rosiglitazone, at the same concentration. The structure‒activity relationship (SAR) of the tested chalcone derivatives statistically supported our in vitro observation [6].

To date, the most common therapy for treating impaired glucose tolerance and T2DM is direct administration of oral antidiabetic medicines. Certain oral antidiabetic drugs can palliate hyperglycemia via promoting insulin secretion, improving insulin sensitivity in tissues, or reducing the rate of carbohydrate absorption from the gastrointestinal tract [3,13]. Among all medications, the American Diabetes Association (ADA) suggests that Metformin, an adenosine monophosphate-activated protein kinase (AMPK) activator, is the optimal first-line drug for treating hyperglycemia [13]. Metformin is used not only in monotherapy but also in combinational therapy with other antidiabetic drugs, such as sulfonylurea, thiazolidinediones (TZDs), dipeptidyl peptidase-4 (DPP-4) inhibitors, glucagon-like peptide-1 (GLP-1) agonists, or insulin [13]. The AMPK pathway plays important roles in mediating blood glucose entering peripheral cells, and inhibiting gluconeogenesis in the liver [14,15,16,17]. Activators of AMPK are proposed to be useful for treating metabolic disorders, and have been a focus of active research and development for the pharmaceutical industry [18,19].

**Figure 1:**
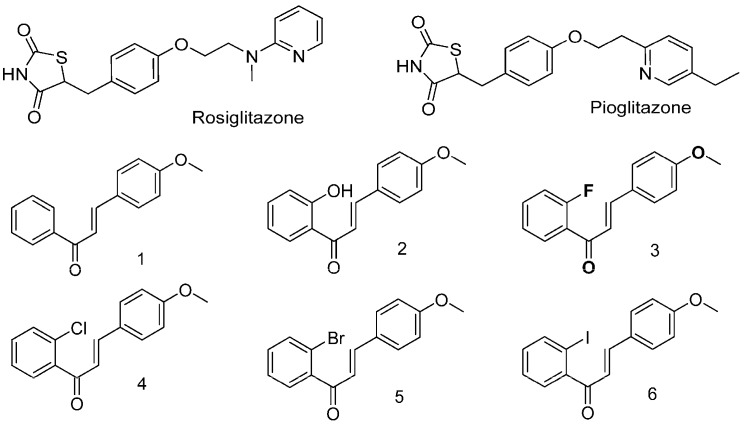
Chemical structures of rosiglitazone, pioglitazone, and chalcone derivatives.

The aims of this study were to confirm the efficacy and to investigate the possible mechanism of action of our candidate chalcone derivatives in antidiabetic models. According to our previous study [6], we selected six lead chalcone derivative compounds (Figure 1) to compare their ability to promote glucose consumption and reduce lipid accumulation, as well as their influence on AMPK activity. Our results suggest that halogen chalcones may serve as novel lead compounds for developing a new category of AMPK-activating agents for the treatment of diabetes.

## 2. Results

### 2.1. Halogen Chalcone Derivatives Promote Glucose Consumption and Reduce Lipid Accumulation in Adipocytes

**Figure 2:**
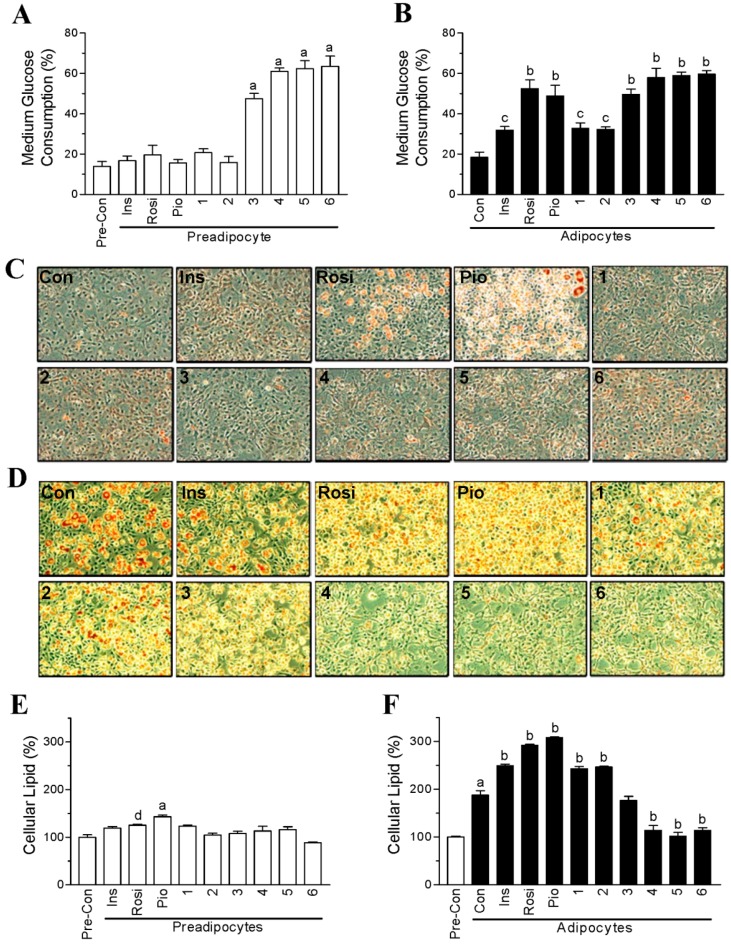
Effects of chalcone derivatives on glucose consumption and lipid accumulation in preadipocytes and adipocytes. 3T3-L1 preadipocytes or adipocytes were incubated with vehicle (0.1% dimethyl sulfoxide; Control), insulin (0.32 µM; Ins), rosiglitazone (30 µg/mL; Rosi), pioglitazone (30 µg/mL; Pio) or 30 µg/mL of chalcone derivatives (compounds **1**–**6**). (**A**) Percentage of medium glucose consumed by the preadipocytes; (**B**) percentage of medium glucose consumed by the adipocytes; (**C**) cellular lipid accumulation in the preadipocytes; (**D**) cellular lipid accumulation in the adipocytes. Red color shows the cellular lipid droplets stained with Oil-Red O; (**E**,**F**) cellular lipid amount was quantified by dissolving the Oil Red O in isopropanol. Data are mean ± SD from three or four independent experiments. ^a^: *p* < 0.001 compared to Pre-Con; ^b^: *p* < 0.001 compared to Con; ^c^: *p* < 0.05 compared to Con; ^d^: *p* < 0.05 compared to Pre-Con. Pre-Con: control for preadipocytes; Con: control for adipocytes. The original magnification of the cell images in panel C and D is 100× under the light microscope.

To evaluate the glucose-lowering activity of compounds **1**–**6** (Figure 1), we tested whether they could promote glucose consumption from the medium of undifferentiated preadipocytes and differentiated adipocytes. After a 24-h treatment, the preadipocytes only consumed approximately 13% of glucose from the medium (Figure 2A). Insulin, rosiglitazone, pioglitazone, compound **1**, and compound **2** did not further promote glucose consumption by the preadipocytes (Figure 2A). In contrast, compounds **3**–**6** further facilitated 30–50% of glucose consumption by the preadipocytes (Figure 2A). The differentiated adipocytes consumed approximately 18% of glucose from the medium (Figure 2B). Insulin, compound **1**, and compound **2** displayed similar efficacy in increasing glucose consumption by approximately 10–15% with the differentiated adipocytes (Figure 2B). However, compounds **3**–**6** significantly increased glucose consumption by 30–40% in differentiated adipocytes, and their efficacy was comparable to that of rosiglitazone and pioglitazone (Figure 2B).

To investigate the influence of compounds **1**–**6** on lipid formation, we stained the cells with Oil-Red O and quantified the amount of cellular lipid. As shown in Figure 2C,E, rosiglitazone and pioglitazone significantly stimulated adipogenesis and increased lipid accumulation in the preadipocytes by 20–50%. In contrast, compounds **1**–**6** had no effect on adipogenesis, even though compounds **3**–**6** increased the glucose consumption. Six days after differentiation, the amount of cellular lipid was increased by approximately 100% in the adipocytes (Figure 2D,F). Insulin, rosiglitazone, pioglitazone, compound **1**, and compound **2** further increased the cellular lipid amount by 50‒100% in the adipocytes (Figure 2D,F). In contrast, compounds **4**–**6** significantly decreased the amount of lipid in the adipocytes by approximately 50% (Figure 2D,F), even though the three compounds were found to facilitate glucose consumption by the adipocytes.

### 2.2. Glucose-Lowering Activities of Halogen Chalcone Derivatives Are Associated with AMPK

**Figure 3:**
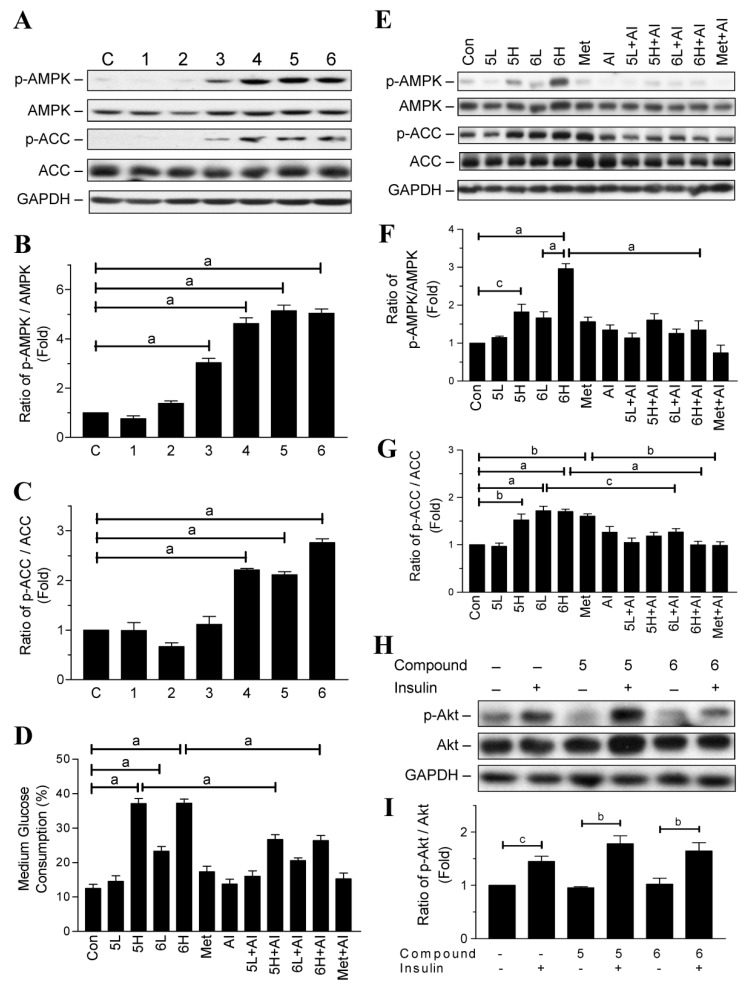
Effects of chalcone derivatives on 5′-adenosine-monophosphate-activated protein kinase (AMPK) and Protein kinase B (Akt) signaling in 3T3-L1 adipocytes. (**A**) 3T3-L1 adipocytes were treated with or without the tested compounds (30 µg/mL) for 24 h, and then phosphorylated and total AMPK and acetyl-CoA carboxylase (ACC) proteins were detected by Western blotting; (**B**,**C**) quantitative results for the blots in panel A; (**D**) 3T3-L1 adipocytes were pre-treated with or without Dorsomorphin (10 µM; AI) for 30 min and then administered with low dosage (15 µg/mL; L) or high dosage (30 µg/mL; H) of compound **5** or **6**, or 30 µg/mL of metformin. The medium glucose consumption was detected after 12 h of incubation and the cells were harvested for protein analysis; (**E**) Western blotting for phosphorylated and total AMPK and ACC proteins; (**F**,**G**) quantitative results for the blots in panel E; (**H**) 3T3-L1 adipocytes were treated with 30 µg/mL of compounds **5** or **6** combined with or without insulin (0.32 µM) for 30 min to observe the phosphorylated and total Akt proteins; (**I**) quantitative results for the blots in panel H. Data are mean ± SD from three independent experiments. ^a^: *p* < 0.001; ^b^: *p* < 0.01; ^c^: *p* < 0.05.

AMPK and insulin signaling are the two main pathways to regulate glucose utilization in adipocytes [15]. Hence, we investigated whether the compounds could influence AMPK, and its downstream metabolic enzyme acetyl-CoA carboxylase (ACC). After 24 h treatment, compounds **3**–**6** significantly increased the phosphorylation of AMPK in the adipocytes, whereas compounds **1** and **2** had no effect on the activation of AMPK (Figure 3A,B). In addition, compounds **4**–**6** significantly increased the phosphorylation of ACC (Figure 3A,C).

To confirm that the glucose-lowering activity of compounds **5** and **6** is via AMPK activation, we treated adipocytes with dorsomorphin (AMPK inhibitor; AI) to observe whether it could reverse the glucose-lowering activity of the two compounds. In agreement with our previous results, after 12 h treatment, compounds **5** and **6** increased glucose consumption by the adipocytes in a dose-dependent manner (Figure 3D). However, treatment with dorsomorphin prevented even high dosages (30 µg/mL) of compounds **5** and **6** from promoting glucose consumption (Figure 3D). Importantly, dorsomorphin itself did not influence the level of glucose consumption in the adipocytes (Figure 3D). As a positive control, metformin seemed to increase glucose consumption, but the increased level did not reach statistical significance (Figure 3D). Together, these results indicate that the glucose-lowering activity of compounds **5** and **6** is, at least in part, via AMPK activation.

As shown in Figure 3E–G, 30 µg/mL of compound **5** (5H) significantly increased the phosphorylation of AMPK and ACC after 12 h treatment. Although dorsomorphin slightly reversed the phosphorylation, it did not reach statistical significance. Similarly, 15 µg/mL of compound **6** (6L) increased AMPK phosphorylation approximately 1.6-fold, but it did not reach statistical significance. Nonetheless, treatment with 15 µg/mL of compound **6** significantly increased ACC phosphorylation. Furthermore, treatment with a high dosage of compound **6** (30 µg/mL, 6H) significantly increased the phosphorylation of AMPK and ACC after 12 h treatment, but this effect was significantly inhibited by treatment with dorsomorphin.

Protein kinase B (Akt) plays a key role in signaling downstream of insulin and facilitates the translocation of glucose transporter 4 (Glut-4) to the cell membrane [15]. To investigate whether compounds **5** and **6** could influence insulin’s action, we treated the adipocytes with 30 µg/mL of compounds **5** and **6** combined with or without 0.32 µM insulin for 30 min to observe the phosphorylation of Akt. As shown in Figure 3H,I, insulin significantly increased Akt phosphorylation in the adipocytes treated with or without compounds **5** and **6**. However, the increased level of phosphorylated Akt did not show a difference among the three groups (Figure 3H,I).

### 2.3. Compounds ***5*** and ***6*** Decrease Cellular Lipids via Inhibition of PPARγ and C/EBPα

**Figure 4:**
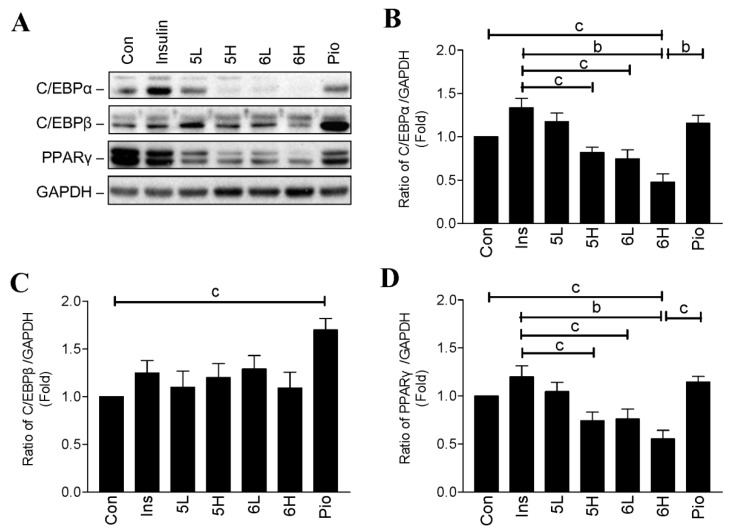
Effects of chalcone derivatives on the expression of CCAAT/enhancer binding protein-α and -β (C/EBPα, C/EBPβ), and peroxisome proliferator activated receptor γ (PPARγ) proteins in 3T3-L1 adipocytes. (**A**) 3T3-L1 adipocytes were treated with or without 0.32 µM of insulin, low dosage (15 µg/mL; L) or high dosage (30 µg/mL; H) of compound **5** or compound **6**, or 30 µg/mL of pioglitazone (Pio) for 24 h. C/EBPα, C/EBPβ, and PPARγ proteins were detected by Western blotting; (**B**–**D**) the quantitative results for the blots in panel A. Data are mean ± SD from three independent experiments. ^a^: *p* < 0.001; ^b^: *p* < 0.01; ^c^: *p* < 0.05.

Peroxisome proliferator activated receptor γ (PPARγ), and CCAAT/enhancer binding protein-α and -β (C/EBPα and C/EBPβ) are key adipogenic transcription factors that regulate differentiation and lipogenesis in adipocytes [14]. Given the effect of compounds **5** and **6** on reducing lipid content (Figure 2D) indicates that these compounds may have influence on these factors. Interestingly, compounds **5** and **6** decreased protein expression of PPARγ and C/EBPα, but not C/EBPβ (Figure 4). In contrast, the cells treated with insulin and pioglitazone maintained high expression levels of PPARγ, C/EBPα, and C/EBPβ, which were similar to those of control adipocytes.

### 2.4. Compounds ***5*** and ***6*** Promote Glucose Consumption in C2C12 Myotubes

**Figure 5:**
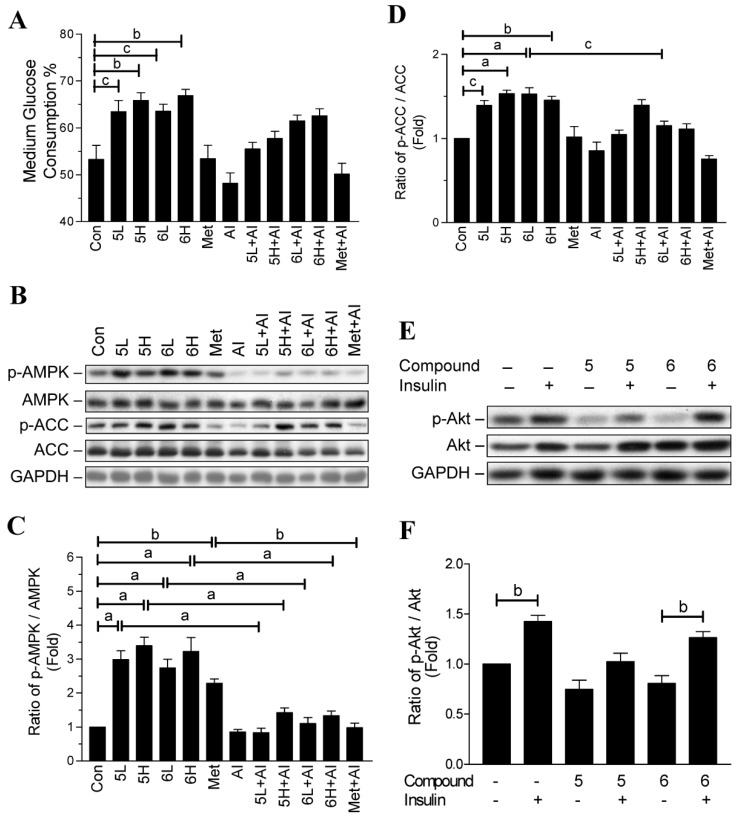
Effects of chalcone derivatives on the glucose consumption, and AMPK and Akt signaling in C2C12 myotubes. (**A**) C2C12 myotubes were pre-treated with or without dorsomorphin (10 µM; AI) for 30 min and then administered a low dosage (15 µg/mL; L) or high dosage (30 µg/mL; H) of compounds **5** or **6**, or 30 µg/mL of metformin. The medium glucose consumption was detected after 12 h of incubation and the cells were harvested for protein analysis; (**B**) Western blotting for phosphorylated and total AMPK and ACC proteins; (**C**,**D**) the quantitative results for the blots in panel B; (**E**) C2C12 myotubes were treated with 30 µg/mL of compounds **5** or **6** combined with or without insulin (0.32 µM) for 30 min, and then the cells were harvest for phosphorylated and total Akt proteins by Western blotting; (**F**) the quantitative results for the blots in panel E. Data are mean ± SD from three independent experiments. ^a^: *p* < 0.001; ^b^: *p* < 0.01; ^c^: *p* < 0.05.

Skeletal muscle is the major site for postprandial blood glucose homeostasis [2,14,15]. Thus, we also tested the antidiabetic activity using a C2C12 myotube model. After 12 h of treatment, C2C12 myotubes consumed approximate 55% of glucose in the medium (Figure 5A). Compounds **5** and **6** significantly increased glucose consumption by the C2C12 myotubes approximately 10% more than the control cells (Figure 5A). However, dorsomorphin reversed the effect of compounds **5** and **6** on promoting glucose consumption, but it did not reach statistical significance (Figure 5A). In addition, metformin had no effect on further promoting glucose consumption by the C2C12 myotubes (Figure 5A).

Next, we determined what effect compounds **5** and **6** had on AMPK signaling in C2C12 myotubes. Compounds **5** and **6**, as well as metformin, significantly increased AMPK phosphorylation after 12 h treatment in the C2C12 myotubes, this effect was significantly reversed by dorsomorphin (Figure 5B,C). Similarly, ACC phosphorylation was significantly increased by compounds **5** and **6**, but dorsomorphin inhibited the phosphorylation, although it did not reach statistical significance (Figure 5B,D). Interestingly, metformin had no effect on ACC phosphorylation (Figure 5B,D).

We then analyzed the influence of compounds **5** and **6** on insulin signaling in C2C12 myotubes. Our results showed that insulin stimulated Akt phosphorylation in the myotubes treated with or without the compounds (Figure 5E,F). However, the increased level of Akt phosphorylation did not show a difference among the three groups, indicating that compounds **5** and **6** did not influence insulin sensitivity in the myotubes.

### 2.5. Compounds ***5*** and ***6*** Prevent Progression of Hyperglycemia and Obesity in HF-Fed Mice

**Figure 6:**
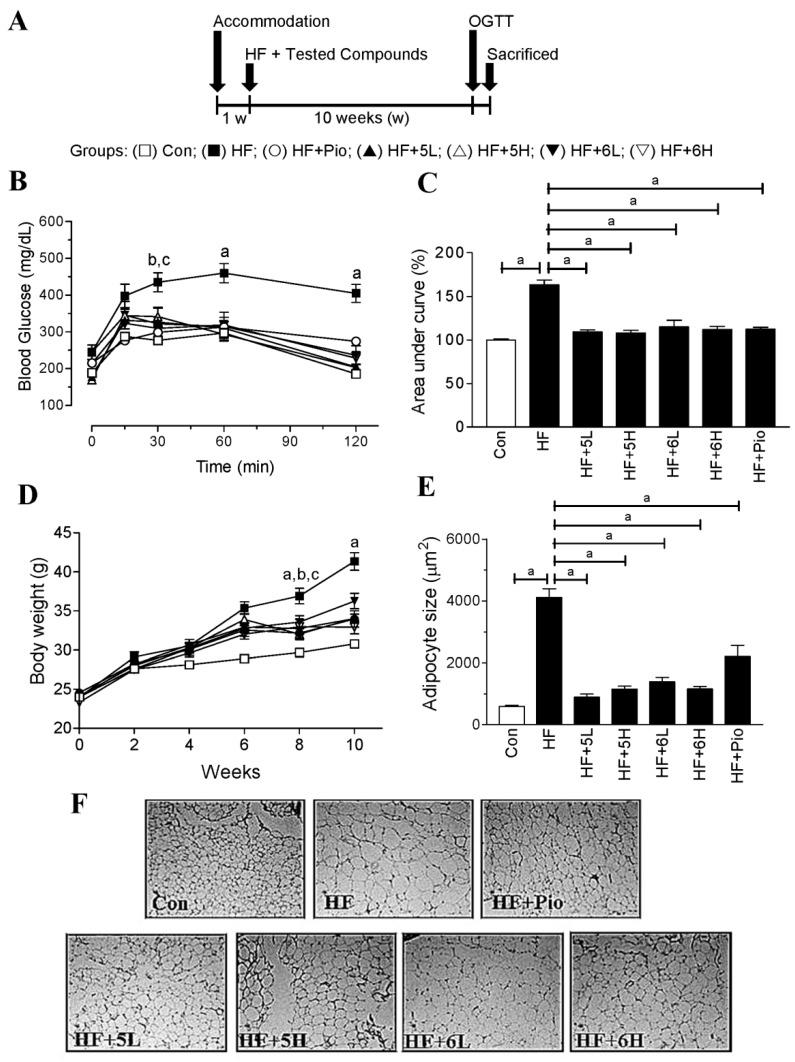
Anti-obesity and antidiabetic effects of compounds 5 and 6 in the HF-fed mice. (**A**) Illustration of the animal protocol; (**B**) results of OGTT in the mice; (**C**) area under curve (AUC) of the OGTT results; (**D**) body weight changes in the mice; (**E**) the quantitative results of the adipocyte size in panel F; (**F**) morphology of the visceral adipose tissues were evaluated by hematoxylin and eosin staining. The original magnification of the tissue images in panel F is 200× under the light microscope. Con: normal-diet-fed; HF: high-fat-diet-fed; HF + 5L: high-fat-diet fed with compound **5** (6.75 mg/kg/day); HF + 5H: high-fat-diet fed with compound **5** (33.75 mg/kg/day); HF + 6L: high-fat-diet fed with compound **6** (6.75 mg/kg/day); HF + 6H: high-fat-diet fed with compound **6** (33.75 mg/kg/day); HF + Pio: high-fat-diet fed with pioglitazone (6.75 mg/kg/day). Data are mean ± SD. In panel B, ^a^: *p* < 0.001, HF group compared with all the other groups; ^b^: *p* < 0.01, HF group compared with Con, HF + 5L, HF + 6H, and HF + Pio; ^c^: *p* < 0.05, HF group compared with HF + 5H, and HF + 6L. ^a^: *p* < 0.001, compared with HF group in panel C and E. In panel D, ^a^: *p* < 0.001, HF group compared with all the other groups; ^b^: *p* < 0.01, HF group compared with HF + 5L, HF + 6H, and HF + Pio; ^c^: *p* < 0.05, HF group compared with HF + 6L. For detailed statistical results of (**B**,**D**) see Table S2.

To confirm the antidiabetic activity of compounds **5** and **6**, we tested their effects on the high-fat diet fed mouse model (preventive model; Figure 6A). As shown in Table 1, the body weight, plasma glucose, and plasma insulin in the HF-fed mice were significantly higher than those in the chow-diet-fed mice. In agreement with the in vitro results, compounds **5** and **6** significantly prevented the elevation of plasma glucose and insulin levels in the mice with HF feeding (Table 1). Furthermore, the results from HOMA-IR (Table 1) and OGTT demonstrated the two compounds significantly prevented insulin resistance and improved glucose tolerance in the mice with HF feeding (Figure 6B,C). Notably, the efficacy of the two compounds in preventing insulin resistance and diabetes was comparable to pioglitazone. Plasma triglyceride (TG) levels remained normal, but total cholesterol (T-CHO) levels were significantly increased in the HF-fed mice (Table 1). Although compounds **5** and **6** demonstrated convincing results regarding diabetes prevention, they were not able to inhibit the elevation in plasma total cholesterol levels induced by HF feeding (Table 1). Compounds **5** and **6** also showed excellent efficacy in inhibiting the body weight gain (Table 1 and Figure 6D). As shown in Figure 6E,F, the cell size of adipocytes in the HF group was enlarged, compared to the control group. However, administration of compounds **5** and **6** could reduce the adipocyte size of the mice with HF feeding.

To test whether these two compounds still could inhibit hyperglycemia in the mice already developed insulin resistance and obesity, we performed an additional animal model (treatment model) in which compounds **5** and **6** were administered after the mice were fed with high fat diet for 12 weeks. The result also demonstrated that these two compounds significantly prevented progression of hyperglycemia and obesity (see Table S1 and Figure S1).

### 2.6. Compounds ***5*** and ***6*** Prevent Progression of Hyperglycemia and Obesity via AMPK Activation in HF-Fed Mice

**Figure 7:**
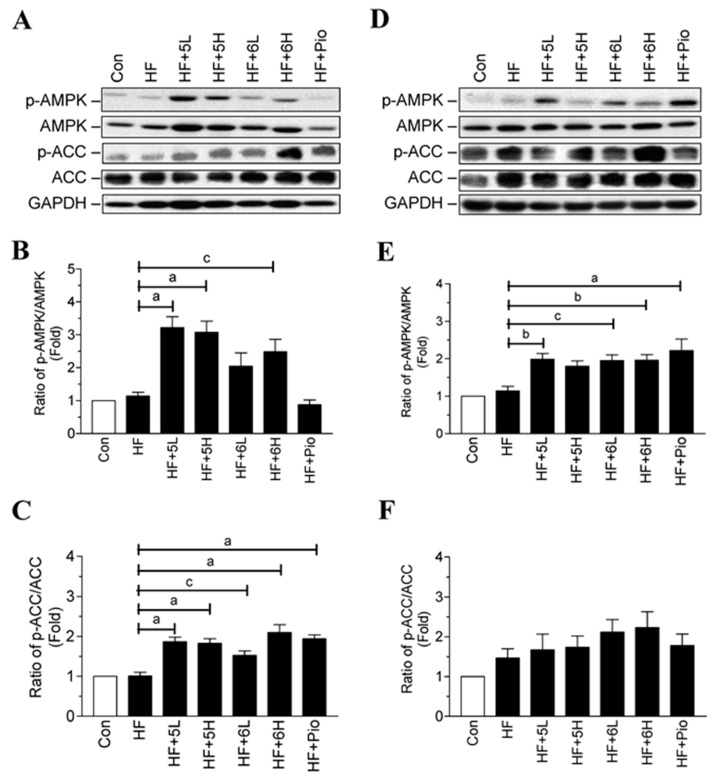
Effects of compounds **5** and **6** on AMPK signaling pathway in the HF-fed mice. (**A**) Proteins from adipose tissues were extracted from the mice, and then phosphorylated and total AMPK and ACC proteins were detected by Western blotting; (**B**,**C**) the quantitative results for the blots in panel A; (**D**) proteins from skeletal muscles were extracted from the mice, and then phosphorylated and total AMPK and ACC proteins were detected by Western blotting; (**E**,**F**) the quantitative results for the blots in panel D. Con: normal-diet-fed; HF: high-fat-diet-fed; HF + 5L: high-fat-diet fed with compound **5** (6.75 mg/kg/day); HF + 5H: high-fat-diet fed with compound **5** (33.75 mg/kg/day); HF + 6L: high-fat-diet fed with compound **6** (6.75 mg/kg/day); HF + 6H: high-fat-diet fed with compound **6** (33.75 mg/kg/day); HF + Pio: high-fat-diet fed with pioglitazone (6.75 mg/kg/day). Data are mean ± SD. ^a^: *p* < 0.001; ^b^: *p* < 0.01; ^c^: *p* < 0.05, compared with HF group.

Our results demonstrate that compounds **5** and **6** may act as AMPK activators and then inhibit ACC in the adipocytes and the C2C12 myotubes. Hence, we explored whether the two compounds could activate AMPK and ACC in mice. As shown in Figure 7A–C, compounds **5** and **6** significantly increased AMPK and ACC phosphorylation in the adipose tissue of the HF-fed mice. Compounds **5** and **6** also increased AMPK and ACC phosphorylation in the skeletal muscle of the HF-fed mice (Figure 7D–F). These in vivo results confirm that compounds **5** and **6** prevent the progression of diabetes and obesity via AMPK activation.

## 3. Discussion

Chalcones are a group of aromatic enones that belong to the flavonoid family. These enones participate in the biosynthetic pathways of flavonoids that form the central core of a variety of important biological compounds and are often responsible for the yellow pigmentation in plants [4,20,21]. Although several natural and synthetic chalcones have been reported concerning their antidiabetic properties, halogen-containing chalcone derivatives have not been fully investigated [6,12,22,23,24,25,26,27,28,29,30,31,32,33,34,35,36,37,38,39,40,41,42]. We reported that some of the halogen-containing chalcone derivatives demonstrated potent effects in promoting glucose consumption in adipocytes [6]. According to the data of a structure‒activity relationship (SAR) analysis from our synthetic chalcone derivatives, substitution (hydrogen, hydroxyl, fluoro, chloro, bromo, and iodo) at position 2 of A-ring significantly affected the antidiabetic activity [6]. In addition, the presence of a methoxy group on the B ring also positively affected the antidiabetic activity [6]. Therefore, we selected compounds **1**–**6** to further investigate their potential in developing antidiabetic medicine.

Our results confirmed that compounds **3**–**6** with halogen substitutions at position 2 of the A-ring significantly promoted the glucose consumption in the media by the pre-adipocytes without influencing of differentiation relative to that of insulin, rosiglitazone, pioglitazone, compound **1** (hydrogen substitution), and compound **2** (hydroxyl substitution). In the differentiated adipocytes, the glucose consumption was significantly increased by the treatments of insulin, rosiglitazone, pioglitazone, and the six compounds. The efficacy of compounds **3**–**6** was approximately twice that of compounds **1** and **2**, indicating that the existence of halogens on the chalcone A-ring could increase the antidiabetic activity. Notably, compounds **4**–**6** increased the glucose consumption of adipocytes, but significantly decreased the cellular lipid droplet accumulation. This phenomenon was different with the cells treated with rosiglitazone and pioglitazone, which facilitated glucose removal from the culture medium, but induced differentiation of preadipocytes and increased cellular lipid accumulation in the differentiated adipocytes. Skeletal muscle is one of the major organs for glucose expenditure [2,15]. Our results also demonstrated that compounds **5** and **6** could facilitate the removal of glucose from the culture medium in the differentiated C2C12 myotubes, suggesting that skeletal muscle may be one of the effective targets for the antidiabetic action of the two compounds.

The tested halogen-containing chalcone derivatives do not exist in natural resources but can be produced by a simple synthetic method [6]. Several of the effective derivatives can be easily crystallized with high yield and purity, in particular compounds **5** and **6**. For this reason, we selected compounds **5** and **6** to confirm their antidiabetic activities in HF-induced type 2 diabetic mouse models. In the preventive model, compounds **5** and **6** could assist in maintaining the same body weight, fasting plasma glucose, plasma insulin, and HOMA-IR levels in mice treated with HF as those in the mice fed a normal diet. In addition, the efficacy of compounds **5** and **6** closely parallels pioglitazone. Unfortunately, the two compounds did not possess the ability to prevent the elevation of plasma cholesterol. In the treatment model, compounds **5** and **6** also significantly attenuated the body weight gain, and the rise in fasting plasma glucose and insulin levels in the mice with pre-existing insulin resistance and obesity induced by 12-week HF feeding (see Figure S1 and Table S1). Furthermore, long-term HF feeding could result in hepatic steatosis and liver injury, but this injury was partially attenuated by the two compounds. The results confirmed that compounds **5** and **6** could prevent obesity, insulin resistance, and diabetes.

AMPK is a fuel-sensing enzyme that is activated by an elevated AMP/ATP ratio, which occurs upon a decrease in a cell’s energy state [18,43,44,45]. When activated, AMPK initiates metabolic and genetic events that restore ATP levels by stimulating processes that generate ATP and inhibiting others that consume energy but are not required for survival [18]. AMPK has been reported to play an important role in cellular energy homeostasis through the stimulation of glucose uptake in adipose tissues and skeletal muscles and the inhibition of lipolysis and lipogenesis in adipocytes [15,18,19,43,44,45]. AMPK mediates these effects through the phosphorylation of metabolic enzymes, such as ACC, the rate-limiting enzyme for fatty acid oxidation [46]. In response to metabolic stress, AMPK is switched on to activate many downstream targets that mediate dramatic changes in cell metabolism, cell growth, and other functions. The use of pharmacological agents to modulate AMPK activity has been exploited therapeutically to treat cardiometabolic diseases [15,47]. According to our findings, the main pharmacological mechanism of our halogen-containing chalcone derivatives is AMPK signaling, which improves insulin resistance, retards diabetes progression, and diminishes fat accumulation. The antidiabetic effect of compounds **5** and **6** is not via activation of insulin signaling because the two compounds were not able to further enhance the phosphorylation of Akt in response to insulin stimulation in neither the 3T3-L1 adipocytes nor the C2C12 myotubes. The mechanism of the two compounds in reducing adiposity may not only be due to AMPK, but could also result from an inhibition of the adipogenic transcriptional factors C/EBPα and PPARγ. In addition to the AMPK pathway, we explored other possible antidiabetic mechanisms of the two compounds. We detected the effect of compounds **5** and **6** on a set of proteins involving in glucose homeostasis: aldose reductase, α-d-glucosidase, glycogen phosphorylase, lipase, dipeptidyl peptidase 4 (DPP4), phosphodiesterases (PDE3, PDE5), protein serine/threonine kinases (AKT1, PRKBA, GSK3B), protein tyrosine kinases (IGF1R, JTK13), protein tyrosine phosphatases (PTPN1, PTP1B), adrenergic β3, glucagon, insulin, potassium channels (K_ATP_, hERG), and purinergic P_2Y_ receptor. The influence on the proteins were measured using enzyme- or radio-ligand binding assays that were performed by a preclinical contract research organization (Ricerca Biosciences, LLC., Taipei, Taiwan). As shown in Table S3, the results of all the tests demonstrated no significant response, indicating that the antidiabetic effect of compounds **5** and **6** was not mediated through these targets. Further investigation is still needed to clarify the pharmacological pathway of the two compounds.

Concerning toxicity, we did not observe any remarkable liver or renal injury after the 10-week (Table 1) and 12-week (Table S1) administrations of compounds **5** and **6** in the mice, according to the GPT and creatinine levels. In addition, the results of the 14-day single-dose acute-toxicity study did not show any toxicity or abnormal clinical signs in the rats (Table S4). The estimated LD 50 is higher than 2000 mg/kg, which is much higher than the effective dosage (6.75 and 33.75 mg/kg) administered to the mice in this study. Taken together, compounds **5** and **6** demonstrate no significant toxicity.

**Figure 8:**
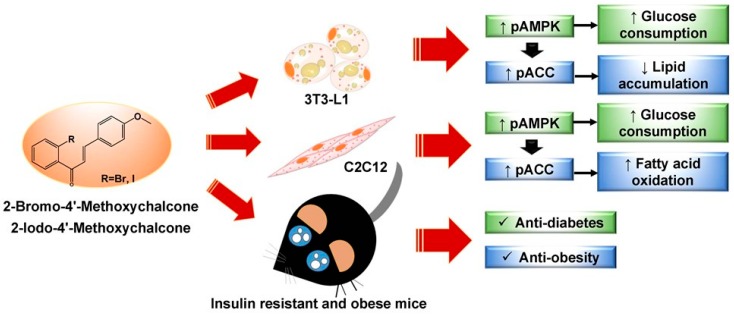
Summary of the antidiabetic and anti-obesity effects of 2-bromo-4′-methoxychalcone and 2-iodo-4′-methoxychalcone.

As shown in Figure 8, we present two novel chalcone derivatives, 2-bromo-4′-methoxychalcone (compound **5**) and 2-iodo-4′-methoxychalcone (compound **6**), that possess excellent antidiabetic and anti-obesity abilities via AMPK activation in adipose tissue and skeletal muscle. The two halogen-containing chalcone derivatives could serve as lead compounds to establish a new category of AMPK activators and to develop medicine for treating diabetes and obesity.

## 4. Materials and Methods

### 4.1. Chemicals

d-glucose, 3-isobutyl-1-methylxanthine, dexamethasone, insulin, ethanol (EtOH), Oil-Red O, propylene glycol, dimethyl sulfoxide (DMSO), pioglitazone, metformin, dorsomorphin (an AMPK inhibitor, AI), and all other chemicals were of analytical grade and obtained from Sigma-Aldrich Inc. (St. Louis, MO, USA). Normal glucose (100 mg/dL) Dulbecco’s Modified Eagle Medium (DMEM; Catalog No.: 12320), penicillin/streptomycin (P/S), fetal bovine serum (FBS), horse serum, and trypsin-EDTA were bought from Invitrogen (Carlsbad, CA, USA). Rosiglitazone used in this experiment did not contain any inactive ingredients, and the pure compound was kindly provided by GlaxoSmithKline, Ltd. (Taipei, Taiwan). Phospho-AMPK, AMPK, phospho-ACC, ACC, phospho-AKT, AKT, PPARγ, C/EBPα, C/EBPβ, and GAPDH antibodies were purchased from Cell Signaling Technology (Danvers, MA, USA).

### 4.2. Synthesis of Compounds

The detailed method for the synthesis of compounds **1**–**6** was described in our previous study [6]. Specifically, compound **1** is the parental 4′-methoxychalcone without any substitution on the A-ring; compound **2** is 2-hydroxy-4′-methoxychalcone; compound **3** is 2-fluoro-4′-methoxychalcone; compound **4** is 2-chloro-4′-methoxychalcone; compound **5** is 2-bromo-4′-methoxychalcone; and compound **6** is 2-iodo-4′-methoxychalcone.

### 4.3. Cell Culture and Differentiation

Adipose tissue and skeletal muscle play major roles in regulating whole-body glucose homeostasis [2,14,15]. Therefore, to pre-screen the antidiabetic activity and possible molecular mechanisms for the tested compounds, we used mouse 3T3-L1 preadipocytes (passage 9–12) and C2C12 myoblasts (passage 10–12) as in vitro models. 3T3-L1 (BCRC#60159) and C2C12 (BCRC#60083) cells were purchased from the Bioresource Collection and Research Center (BCRC, Food Industry Research and Development Institute, Taiwan, R.O.C., Hsinchu, Taiwan). The cells (5 × 10^5^) were seeded on six-well plates and maintained in 3 mL of normal glucose (100 mg/dL) DMEM with 10% (*v*/*v*) FBS and 1% P/S in a humidified atmosphere of 95% air and 5% CO_2_ at 37 °C. After reaching 100% confluence, the cells were kept under incubation for additional 48 h. For differentiation, 3T3-L1 cells were cultured with differentiation medium (DMEM containing 10% FBS, 450 mg/dL d-glucose, 0.32 µM insulin, 0.5 mM 3-isobutyl-1-methylxanthine, and 1 µM dexamethasone) for two days and then changed to DMEM containing 10% FBS and 450 mg/dL d-glucose. Similarly, C2C12 cells were cultured in a differentiation medium (DMEM containing 450 mg/dL d-glucose and 2% horse serum) for four days, and the differentiated cells formed myotubes.

### 4.4. Detection of Glucose Consumption

To detect the glucose-lowering activity, 3 mL of DMEM containing 450 mg/dL d-glucose with or without the administration of the tested compounds were added to the undifferentiated 3T3-L1 pre-adipocytes, differentiated 3T3-L1 adipocytes or differentiated C2C12 myotubes. After 24 h, the glucose concentration in the medium was determined using a Roche Cobas Integra 400 Chemistry Analyzer (Roche Diagnostics, Taipei, Taiwan). The glucose-lowering activity was defined by calculating the percentage of glucose consumed from the culture medium [48]. The cells incubated with vehicle (DMSO) containing without the tested compounds were assigned as control groups. Insulin (0.32 µM), metformin (30 µg/mL), pioglitazone (30 µg/mL), and rosiglitazone (30 µg/mL) were used as positive controls to confirm whether our in vitro models were sufficient to measure the glucose-lowering activity. Compound **1**–**6** were dissolved in DMSO to make 50 μg/μL stock solutions, and then further diluted in DMSO for experimentation. The final concentrations of compound **1**–**6** and DMSO in the medium were 30 µg/mL and 0.1%, respectively.

### 4.5. Oil-Red O Staining

To investigate whether the compounds could induce differentiation, we added them to the non-differentiated preadipocytes when the cell density reached 100% confluence. In order to observe the influence on lipid formation, we treated the adipocytes with the compounds in DMEM containing 450 mg/dL glucose for four days, with the medium and compounds being changed every other day. Then, the cells were washed twice with phosphate buffered saline (PBS) and fixed with 4% paraformaldehyde for 30 min at room temperature. The fixed cells were stained with 0.5% Oil-Red O that was dissolved in propylene glycol. After 1 h, the cells were washed with PBS, and then lipid droplets were observed under a light microscope. Stained lipid droplets were dissolved in isopropanol, and quantified by a spectrophotometer with absorbance at OD 490 nm.

### 4.6. Animal Studies

A type 2 diabetic model conducted by simply feeding a high-fat diet to a non-obese, non-diabetic C57BL/6J mouse strain was initially developed in Japan. Although the model requires longer feeding times to develop hyperglycemia, it closely resembles the natural disease progression in humans [48,49,50].

Eight-week-old male C57BL/6J mice were obtained from BioLASCO Technology (Charles River Taiwan Ltd., Taipei, Taiwan). All mice received standard animal care in accordance with the Guide for the Care and Use of Laboratory Animals (National Institutes of Health, Bethesda, MD, USA). The experiments were approved and supervised by the Institutional Animal Care and Use Committee (IACUC) of Kaohsiung Medical University (IACUC permission numbers: 98049, approved on 26 August 2009). All efforts were made to minimize the suffering of animals and to reduce the number of animals used. The mice were caged in an air-conditioned animal facility at 22 °C on a 12 h light/dark cycle and were given free access to water and food. They were fed either a normal chow diet consisting of 11% fat (as a percentage of total kcal), 65% carbohydrate, and 24% protein (Maintenance diet 1320, Altromin Spezialfutter GmbH & Co. KG, Lage, Germany), or a high-fat diet (HF) consisting of 45% fat, 35% carbohydrate, and 20% protein (D12451, Research Diets, Inc., New Brunswick, NJ, USA). Seven days post-arrival, mice were randomly divided into seven groups: (i) normal-chow-diet fed control mice (Con); (ii) HF fed mice (HF); (iii) HF-fed mice with (6.75 mg/kg/day) pioglitazone (HF + Pio); (iv) HF-fed mice with low dose (6.75 mg/kg/day) compound **5** treatment (HF + 5L); (v) HF-fed mice with high dose (33.75 mg/kg/day) compound **5** treatment (HF + 5H); (vi) HF fed mice with low dose (6.75 mg/kg/day) compound **6** treatment (HF + 6L); and (vii) HF fed mice with high dose (33.75 mg/kg/day) compound **6** treatment (HF + 6H). In preventive model, the administration of pioglitazone, compound **5**, and compound **6** via gastric gavage was begun at day one of HF feeding. Pioglitazone, compound **5**, and compound **6** were resuspended in normal saline. The dose of 6.75 mg/kg/day used in the mice was determined according to the human recommended maximum dose (45 mg/day) of pioglitazone by multiplying 9 (converting factor [51]) and then divided by 60 kg (45 × 9/60 = 6.75 mg/kg/day).

Throughout the experiment, body weight and blood glucose were monitored every two weeks. Blood glucose from the tail tip was detected by an ACCU-CHEK blood glucose meter (Roche Diagnostics, Taipei, Taiwan). The mice were sacrificed after administering the compounds for 10 or 12 weeks. After fasting overnight, the mice were anesthetized by intraperitoneal injection with Zoletil (160 mg/kg) (Virbac, Carros, France). Blood samples were collected from the hearts at the time of sacrifice, centrifuged at 3000 rpm for 15 min, and then the supernatant plasma were stored in a −20 °C freezer. A Roche Cobas Integra 400 Chemistry Analyzer (Roche Diagnostics, Taipei, Taiwan) was applied to obtain the biochemistry data, including fasting plasma glucose, triglyceride, total cholesterol, alanine aminotransferase, and creatinine. Plasma insulin was detected by ELISA kits (Crystal Chem Inc., Elk Grove Village, IL, USA). The homeostasis model assessment of insulin resistance (HOMA-IR) was calculated using the following formula: fasting blood glucose (mg/dL) × fasting insulin (μU/mL)/405.

### 4.7. Oral Glucose Tolerance Test

After administering the compounds to the mice for 10 weeks, we performed oral glucose tolerance test (OGTT). The mice were starved for 5–7 h, and then the basal (0 min) blood glucose concentrations were measured before the administration of 2.0 g/kg glucose via gastric gavage. The following blood samples from the tail tips were taken at 15, 30, 60, and 120 min. Glucose concentration was measured using an ACCU-CHEK blood glucose meter, and area under curve (AUC) of OGTT was calculated by GraphPad Prism 5 (GraphPad Software, La Jolla, CA, USA).

### 4.8. SDS-PAGE and Western Blotting

We used sodium dodecyl sulfate-polyacrylamide gel electrophoresis (SDS-PAGE) and Western blotting to analyze the expression levels of the target proteins. Briefly, the cells were lysed in M-PER Mammalian Protein Extraction Reagent (Thermo Fisher Scientific, Inc., Waltham, MA, USA), supplemented with Complete Protease Inhibitor Cocktail (Roche Applied Science, Penzberg, Germany). Protein concentration was measured using the Quant-iT Protein assay kit (Invitrogen). For analysis of each specific protein, the cell lysate was loaded and separated on 10% SDS-PAGE gels. After transfer to polyvinylidene fluoride (PVDF) membranes, proteins of interest were detected using the appropriate antibodies. The blots were scanned and quantified using the ImageJ software (National Institutes of Health).

### 4.9. Statistical Analysis

All data are presented as mean ± SD. Data were analyzed by one-way analysis of variance (ANOVA) followed by Bonferroni’s Multiple Comparison Test using GraphPad Prism 5 (GraphPad Software, La Jolla, CA, USA). Differences were considered significant at *p* < 0.05.

## Figures and Tables

**Table 1:** Biochemical data of the mice in preventive model.

	Con	HF	HF + 5L	HF + 5H	HF + 6L	HF + 6H	HF + Pio
	(*n* = 13)	(*n* = 9)	(*n* = 10)	(*n* = 10)	(*n* = 8)	(*n* = 10)	(*n* = 6)
Weight (g)	30.8 ± 0.3	41.3 ± 1.1 ^a^	34.0 ± 0.3 ^c,d^	34.1 ± 0.5 ^c,d^	36.3 ± 1.1 ^a,d^	32.9 ± 0.9 ^d^	34.0 ± 1.0 ^d^
Fas-Glu	260.5 ± 10.6	316.7 ± 18.1 ^c^	191.4 ± 9.8 ^b,d^	221.9 ± 13.7 ^d^	236.1 ± 9.4 ^e^	235.2 ± 16.2 ^d^	245.5 ± 16.7 ^f^
Insulin	66.4 ± 6.6	127.3 ± 19.0 ^c^	51.6 ± 30.5 ^f^	48.1 ± 13.4 ^f^	60.0 ± 18.4 ^f^	35.8 ± 10.9 ^e^	41.9 ± 18.3 ^f^
HOMA-IR	39.4 ± 2.7	105. 7 ± 13.5 ^a^	27.8 ± 13.1 ^d^	25.9 ± 7.5 ^d^	35.3 ± 11.8 ^d^	23.2 ± 8.3 ^d^	25.6 ± 11.9 ^d^
T-CHO	79.3 ± 4.1	141.6 ± 10.1 ^a^	129.6 ± 8.6 ^a^	123.2 ± 8.3 ^a^	161.2 ± 9.2 ^a^	144.8 ± 5.8 ^a^	127.2 ± 8.4 ^b^
TG	68.3 ± 6.3	57.8 ± 5.5	70.7 ± 5.7	65.5 ± 4.1	62.3 ± 3.4	54.9 ± 3.0	55.7 ± 3.7
GPT	47.6 ± 2.9	46.7 ± 3.4	43.8 ± 3.7	42.0 ± 2.9	59.1 ± 5.2	46.3 ± 4.0	51.2 ± 4.3
Crea	0.25 ± 0.05	0.16 ± 0.02	0.18 ± 0.02	0.19 ± 0.01	0.19 ± 0.01	0.18 ± 0.01	0.19 ± 0.01

Values are mean ± SD. Data were analyzed by one-way ANOVA followed by the Bonferroni’s Multiple Comparison test. Con: normal-diet-fed; HF: high-fat-diet fed; HF + 5L: high-fat-diet fed compound **5** (6.75 mg/kg/day); HF + 5H: high-fat-diet fed with compound **5** (33.75 mg/kg/day); HF + 6L: high-fat-diet fed with compound **6** (6.75 mg/kg/day); HF + 6H: high-fat-diet fed with compound **6** (33.75 mg/kg/day); HF + Pio: high-fat-diet fed with pioglitazone (6.75 mg/kg/day); Fas-Glu: fasting-glucose (mg/dL); T-CHO: total cholesterol (mg/dL); TG: triglyceride (mg/dL); GPT: alanine aminotransferase (U/L); Crea: creatinine (mg/dL); ^a^: *p* < 0.001 compared to control group (Con); ^b^: *p* < 0.01 compared to Con; ^c^: *p* < 0.05 compared to Con; ^d^: *p* < 0.001 compared to high-fat-diet-fed group (HF); ^e^: *p* < 0.01 compared to HF; ^f^: *p* < 0.05 compared to HF.

## Supplementary Materials

Supplementary materials can be found at http://www.mdpi.com/1422-0067/19/9/2763/s1.

## Abbreviations

AMPK	5′-adenosine-monophosphate-activated protein kinase
ACC	acetyl-CoA carboxylase 1 ACC
Akt	Protein kinase B
PPARγ	Peroxisome proliferator activated receptor γ
C/EBP	CCAAT/enhancer binding protein
