# Supplementary material for: 2-Bromo-4′-methoxychalcone and 2-Iodo-4′-methoxychalcone Prevent Progression of Hyperglycemia and Obesity via 5′-Adenosine-Monophosphate-Activated Protein Kinase in Diet-Induced Obese Mice
Source: Int J Mol Sci. 2018 Sep 14;19(9):2763. doi: 10.3390/ijms19092763 (PMC6163633; doi:10.3390/ijms19092763)
Supplement: Supplementary file 1 [file ijms-19-02763-s001.pdf]

Supplementary Materials

Compounds 5 and 6 prevent progression of hyperglycemia and obesity in HF-fed mice

In the treatment mice model, compounds 5 and 6 was given to mice that had already developed obesity and insulin resistance. The mice were fed with HF for 12 weeks to induce obesity and impaired glucose tolerance. Then, the obese mice were divided into groups and were either given pioglitazone, compound 5, compound 6 or vehicle daily for another 12 weeks. At the end of the treatments, pioglitazone and the two compounds significantly reduced body weight gain of the obese mice (Table S1 and Fig. S1D). The results of OGTT and AUC showed a considerable improvement in glucose tolerance in the obese mice treated with pioglitazone and the two compounds (Fig. S1B, C). Moreover, after 24 weeks of HF feeding, the fasting plasma glucose level in the obese mice was approximately 100 mg/dl higher than that in the chow-diet-fed mice. Pioglitazone and the two compounds significantly inhibited the elevation of fasting plasma glucose level in the obese mice (Table S1).

In addition, plasma insulin level and HOMA-IR were significant lower in the obese mice treated with pioglitazone, compounds 5 or 6 (Table S1), indicating an improvement of insulin resistance after the treatments. As shown in Fig. S1F, adipocyte size in the HF group were enlarged compared to that in the control group. Relative to the HF group, administration of compounds 5 and 6 could reduce the adipocyte size, but this was without statistical significance (Fig. S1E, F).

After 24 weeks HF feeding, plasma triglyceride (TG) levels did not show an increase, but plasma total cholesterol (T-CHO) levels were significantly increased in the obese mice (Table S1). Again, compounds 5 and 6 did not inhibit the elevation of plasma total cholesterol in the obese mice (Table S1). In addition, long-term HF feeding resulted in an elevation of plasma alanine aminotransferase (GPT) levels, indicating a damage of liver function in the obese mice (Table S1). Compounds 5 and 6 significantly reduced GPT levels, suggesting that the two compounds could improve the liver function of the obese mice (Table S1). Although the animal numbers in treatment model were only 3 to 6 in each group, the results still demonstrated that compounds 5 and 6 significantly improved glucose intolerance, and insulin resistance in the severe obese mice.

Table S1. Biochemical data of the mice in treatment model.

	**Con (n=3)**	**HF (n=4)**	**HF+5L (n=6)**	**HF+5H (n=6)**	**HF+6L (n=4)**	**HF+6H (n=4)**	**HF+Pio (n=3)**
Weight (g)	34.5±0.8	53.35±1.09 [a]	46.5±0.9 [a,d]	49.30±0.80 [a]	49.2±0.9 [a]	48. 5±1.3 [a,f]	48.5±0.9 [a]
Fas-Glu	249.8±3.4	367.6±12.1 [a]	272.8±9.8 [d]	278.0±11.3 [d]	295.4±12.6 [e]	293.6±11.9 [e]	280.2±7.0 [d]
Insulin	52.4±4.8	181.9±2.4 [a]	108.2±16.1 [f]	178.2±5.6 [a]	138.3±29.4 [c]	110.3±12.2 [f]	153.9±2.6 [b]
HOMA-IR	32.4±3.4	164.9±3.4 [a]	73.2±11.7 [d]	121.2±3.9 [a,f]	100.2±21.0 [b, e]	80.7±11.1 [d]	106.3±0.8 [b,f]
T-CHO	86.9±1.6	194.4±10.9 [a]	192.5±10.0 [a]	209.6±6.8 [a]	199.5±7.3 [a]	174.2±10.6 [a]	185.7±4.9 [a]
TG	76.3±26.8	70.8±14.4	67.96±5.18	60.7±5.2	60.2±9.1	56.5±7.2	43.0±3.4
GPT	73.0±7.9	224.4±17.2 [a]	127.6±23.2 [e]	163.8±13.4 [c]	128.1±18.6 [f]	99.1±16.3 [e]	181.7±10.3 [c]
Crea	0.22±0.03	0.24±0.02	0.23±0.02	0.24±0.05	0.20±0.02	0.19±0.02	0.22±0.01

*Values are mean ± SD. Data were analyzed by one-way ANOVA followed by the Bonferroni's Multiple Comparison test.

*Fas-Glu: fasting-glucose (mg/dL); T-CHO: total cholesterol (mg/dL); TG: triglyceride (mg/dL); GPT: alanine aminotransferase (U/L); Crea: creatinine (mg/dL); a: $p<0.001$ compared to control group (Con); b: $p<0.01$ compared to Con; c: $p<0.05$ compared to Con; d: $p<0.001$ compared to high-fat-diet-fed group (HF); e: $p<0.01$ compared to HF; f: $p<0.05$ compared to HF.

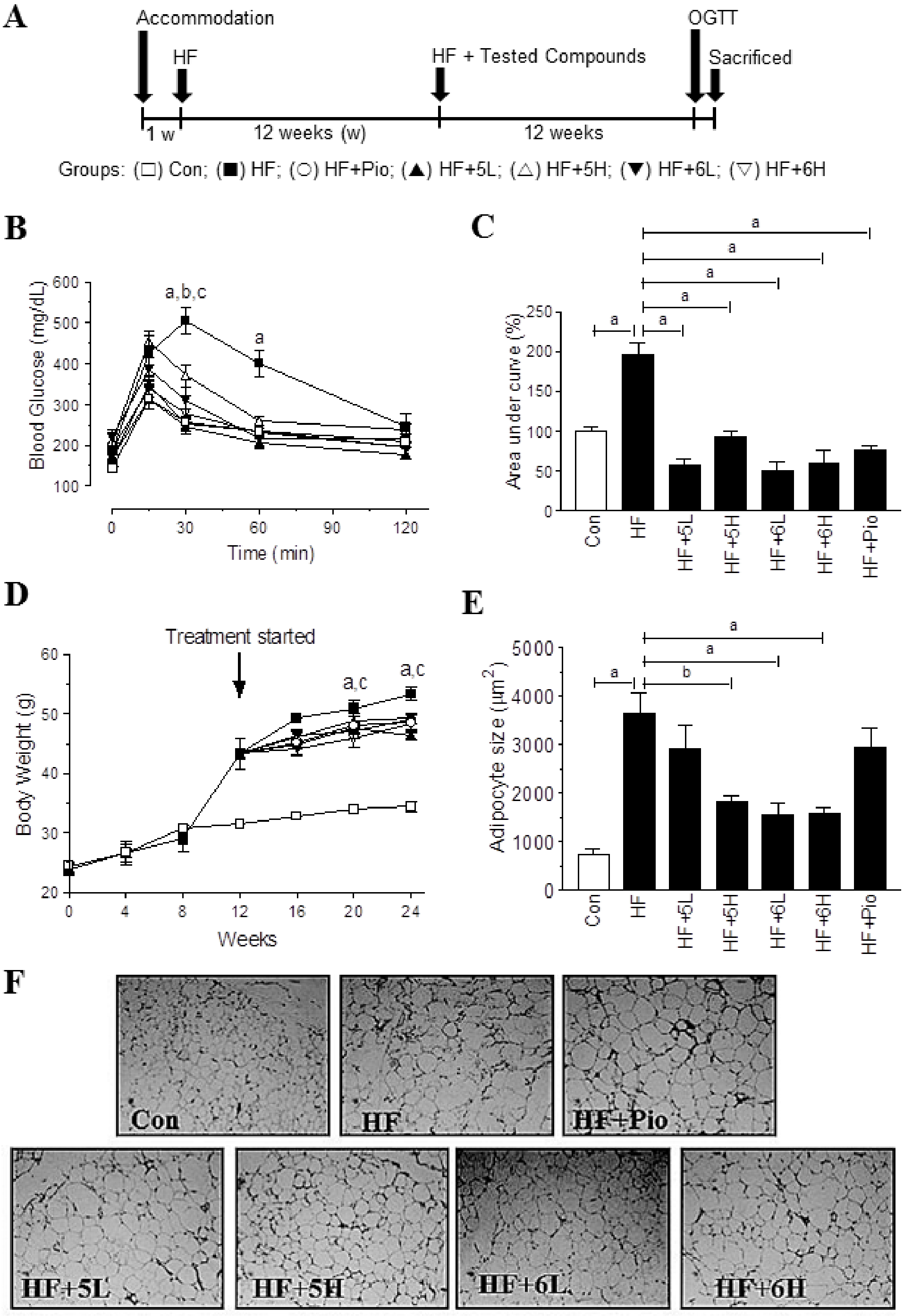

Figure S1. Anti-obese and anti-diabetic effects of compounds 5 and 6 in the treatment mouse model. (A) Illustration of the animal protocol. (B) Results of OGTT in the mice. (C) Area under curve (AUC) of the OGTT results. (D) Body weight changes in the mice. (E) The quantitative results of the adipocyte size in panel F. (F) Morphology of the visceral adipose tissues were evaluated by hematoxylin and eosin staining. Data are mean ± SD. a: $p<0.001$, compared with HF group in panel C and E. The detail statistical results of (B) and (D) please see the supplementary data. Con: normal-diet-fed; HF: high-fat-diet fed; HF+Pio: high-fat-diet fed with pioglitazone (6.75 mg/kg/day); HF+5L: high-fat-diet fed compound 5 (6.75 mg/kg/day); HF+5H: high-fat-diet fed with compound 5 (33.75 mg/kg/day); HF+6L: high-fat-diet fed with compound 6 (6.75 mg/kg/day); HF+6H: high-fat-diet fed with compound 6 (33.75 mg/kg/day).

Table S2. Statistic p values for figure 6 and figure S1.

**Figure 6B**	0 min	15 min	30 min	60 min	120 min		
Con vs HF	p < 0.01	p < 0.05	p < 0.01	p < 0.001	p < 0.001		
HF vs HF+5L	p < 0.001	p > 0.05	p < 0.01	p < 0.001	p < 0.001		
HF vs HF+5H	p < 0.001	p > 0.05	p < 0.05	p < 0.001	p < 0.001		
HF vs HF+6L	p > 0.05	p > 0.05	p < 0.05	p < 0.001	p < 0.001		
HF vs HF+6H	p > 0.05	p > 0.05	p < 0.01	p < 0.001	p < 0.001		
HF vs HF+Pio	p > 0.05	p < 0.01	p < 0.01	p < 0.001	p < 0.001		
**Figure 6D**	0 week	2 week	4 week	6 week	8 week	10 week	
Con vs HF	p > 0.05	p > 0.05	p > 0.05	p < 0.001	p < 0.001	p < 0.001	
HF vs HF+5L	p > 0.05	p > 0.05	p > 0.05	p > 0.05	p < 0.01	p < 0.001	
HF vs HF+5H	p > 0.05	p > 0.05	p > 0.05	p > 0.05	p < 0.001	p < 0.001	
HF vs HF+6L	p > 0.05	p > 0.05	p > 0.05	p > 0.05	p < 0.05	p < 0.001	
HF vs HF+6H	p > 0.05	p > 0.05	p > 0.05	p < 0.01	p < 0.01	p < 0.001	
HF vs HF+Pio	p > 0.05	p > 0.05	p > 0.05	p > 0.05	p < 0.01	p < 0.001	
**Figure S1B**	0 min	15 min	30 min	60 min	120 min		
Con vs HF	p > 0.05	p > 0.05	p < 0.001	p < 0.001	p > 0.05		
HF vs HF+5L	p > 0.05	p > 0.05	p < 0.001	p < 0.001	p < 0.05		
HF vs HF+5H	p > 0.05	p > 0.05	p < 0.05	p < 0.001	p > 0.05		
HF vs HF+6L	p > 0.05	p > 0.05	p < 0.01	p < 0.001	p > 0.05		
HF vs HF+6H	p > 0.05	p > 0.05	p < 0.001	p < 0.001	p > 0.05		
HF vs HF+Pio	p > 0.05	p > 0.05	p < 0.001	p < 0.001	p > 0.05		
**Figure S1D**	0 week	4 week	8 week	12 week	16 week	20 week	24 week
Con vs HF	p > 0.05	p > 0.05	p > 0.05	p < 0.001	p < 0.001	p < 0.001	p < 0.001
HF vs HF+Pio					p < 0.05	p > 0.05	p < 0.05
HF vs HF+5L					p < 0.01	p > 0.05	p < 0.001
HF vs HF+5H					p < 0.05	p > 0.05	p < 0.05
HF vs HF+6L					p > 0.05	p > 0.05	p < 0.05
HF vs HF+6H					p < 0.01	p < 0.05	p < 0.05

Table S3. Biochemical assays of alternative anti-diabetic pathways

	% of stimulation (+) or inhibition (-)	
Targets	Compound 5	Compound 6
Aldose Reductase	+1	+3
α-D-Glucosidase	+2	+8
Glycogen Phosphorylase	+3	+5
Lipase	-3	-2
Peptidase, Dipeptidyl Peptidase 4, DPP4 (DPP IV)	+3	+10
Phosphodiesterase PDE3	-6	-13
Phosphodiesterase PDE5	+30	+31
Protein Serine/Threonine Kinase, AKT1 (PRKBA)	+19	+10
Protein Serine/Threonine Kinase, GSK3B	+20	+21
Protein Tyrosine Kinase, IGF1R (JTK13)	-4	-5
Protein Tyrosine Phosphatase, PTPN1 (PTP1B)	-1	-2
Adrenergic β3	+7	+8
Glucagon	-11	-8
Insulin	+3	+18
Potassium Channel [K_{AKT}]	+8	-2
Potassium Channel hERG	+22	+9
Purinergic P_{2Y}	+17	+23

Experiments are performed in duplicate for each compound. The studies used 10 μM of the test compounds, and significant responses were judged by the criteria of the assays. The assays that showed ≥ 50% inhibition or stimulation were considered to be significant responses.

No significant toxicity induced by compounds 5 and 6

We did not observe any kidney or liver injury induced by compounds 5 and 6 after daily oral administration for 10 weeks (prevention model) or 12 weeks (treatment model) at the dosages of 6.75 and 33.75 mg/kg/day. For example, the plasma creatinine levels were no difference among the control, HF and treatment groups in both experimental models (Tables 1 and S1). The GPT levels also showed no difference among the control, HF and treatment groups in the prevention model (Table 1). In the treatment model, the GPT levels in the compound 5 and compound 6 groups were higher than that of the control group. However, the high GPT levels were due to the effect of HF feeding and aging rather than because of the two compounds (Table S1).

A 14-day single dose acute toxicity study was carried out to evaluate the possible adverse effects of compounds 5 and 6. This study is performed by a preclinical contract research organization (Level Biotechnology INC., Taiwan). The animal facility has been accredited by Association for Assessment and Accreditation of Laboratory Animal Care (AAALAC) International. In the study, Sprague-Dawley (SD) rats were randomly assigned to four groups (0, 50, 300, and 2000 mg/kg) with 6 males and 6 females in each group before dosing. The clinical observation was carried out for 14 days after oral administration of the two compounds. Examinations were conducted on the rats with respect to mortality, mean body weights, mean body weight gains, clinical signs, and gross necropsy findings.

The results from the 14-day single dose acute toxicity study showed that compounds 5 and 6 administered to SD rats *via* a single oral administration did not cause death at the designated dosages of 0, 50, 300, and 2000 mg/kg (Table S4). The mean body weights and mean body weight gains did not show significant differences between the control and treatment groups on Days 1, 4, 8, and 15 ($p > 0.05$). The estimated lethal dose 50 (LD 50) were higher than 2000 mg/kg in both genders. In male and female rats, no abnormal clinical signs and gross necropsy findings were noted during the study period (Table S4).

Table S4. Acute toxicity studies in rats.

Examinations	Gender	Dose (mg/kg)	Compound 5 Days 1	Compound 5 Days 2~7	Compound 5 Days 8~14	Compound 5 Total Incidence (n/n)	Compound 6 Days 1	Compound 6 Days 2~7	Compound 6 Days 8~14	Compound 6 Total Incidence (n/n)
Mortality (Number of Death)	Male	0 [a]	0	0	0	0/6 [b]	0	0	0	0/6 [b]
		50	0	0	0	0/6 [b]	0	0	0	0/6 [b]
		300	0	0	0	0/6 [b]	0	0	0	0/6 [b]
		2000	0	0	0	0/6 [b]	0	0	0	0/6 [b]
	Female	0 [a]	0	0	0	0/6 [b]	0	0	0	0/6 [b]
		50	0	0	0	0/6 [b]	0	0	0	0/6 [b]
		300	0	0	0	0/6 [b]	0	0	0	0/6 [b]
		2000	0	0	0	0/6 [b]	0	0	0	0/6 [b]
Clinical Observations (Abnormality Incidence [c])	Male	0 [a]	0/6	0/6	0/6	0/6 [d]	0/6	0/6	0/6	0/6 [d]
		50	0/6	0/6	0/6	0/6 [d]	0/6	0/6	0/6	0/6 [d]
		300	0/6	0/6	0/6	0/6 [d]	0/6	0/6	0/6	0/6 [d]
		2000	0/6	0/6	0/6	0/6 [d]	0/6	0/6	0/6	0/6 [d]
	Female	0 [a]	0/6	0/6	0/6	0/6 [d]	0/6	0/6	0/6	0/6 [d]
		50	0/6	0/6	0/6	0/6 [d]	0/6	0/6	0/6	0/6 [d]
		300	0/6	0/6	0/6	0/6 [d]	0/6	0/6	0/6	0/6 [d]
		2000	0/6	0/6	0/6	0/6 [d]	0/6	0/6	0/6	0/6 [d]
Body Weights (g) (Mean ± SD, n = 6)			Day 1		Day 15		Day 1		Day 15	
	Male	0 [a]	291.9± 9.5		371.8±19.1		303.5± 7.0		385.9±11.6	
		50	288.8± 7.1		369.5±17.4		306.8± 8.0		378.5±12.7	
		300	292.9± 8.0		366.5±23.2		302.8±10.8		386.9±18.9	
		2000	290.1±10.3		374.4±23.0		297.4±10.4		368.5±12.2	
	Female	0 [a]	209.8±4.7		246.3±15.0		214.7± 6.3		249.8±13.8	
		50	209.4±5.7		244.9±20.4		217.6± 7.1		245.1±14.6	
		300	207.6±9.3		240.9±18.5		217.6± 3.7		250.5±12.9	
		2000	210.3±6.0		239.2± 7.2		218.3± 7.8		250.2±16.1	
Gross Necropsy Findings (Abnormality Incidence [e])			Seminal Vessels		Testes & Epididymides		Seminal Vessels		Testes & Epididymides	
	Male	2000	0/6		0/6		0/6		0/6	
	Female	2000	0/6		0/6		0/6		0/6	

[a] Vehicle control: 100% pure olive oil.

[b] Total number of found dead or moribund animals / Total number of animals.

[c] Number of animals with observable sign / Number of animals alive.

[d] Total number of animals with observable sign / Total number of animals examined.

[e] Animal numbers of gross necropsy findings / Animal numbers of gross necropsy examinations.
